# Phytoremediation: A Novel Approach of Bast Fiber Plants (Hemp, Kenaf, Jute and Flax) for Heavy Metals Decontamination in Soil—Review

**DOI:** 10.3390/toxics11010005

**Published:** 2022-12-20

**Authors:** Fera Nony Cleophas, Nur Zaida Zahari, Pavitra Murugayah, Sahibin Abd Rahim, Ahmad Norazhar Mohd Yatim

**Affiliations:** 1 Environmental Science Programme, Faculty of Science & Natural Resources, Universiti Malaysia Sabah, UMS Road, Kota Kinabalu 88400, Sabah, Malaysia; 2 Small Islands Research Center, Universiti Malaysia Sabah, UMS Road, Kota Kinabalu 88400, Sabah, Malaysia

**Keywords:** phytoremediation, bast fiber plants, heavy metals, hemp, kenaf, jute, Flax, soil

## Abstract

Heavy metal pollution in the environment is a major concern for humans as it is non-biodegradable and can have a lot of effects on the environment, humans as well as plants. At present, a solution to this problem is suggested in terms of a new, innovative and eco-friendly technology known as phytoremediation. Bast fiber plants are typically non-edible crops that have a short life cycle. It is one of the significant crops that has attracted interest for many industrial uses because of its constant fiber supply and ease of maintenance. Due to its low maintenance requirements with minimum economic investment, bast fiber plants have been widely used in phytoremediation. Nevertheless, these plants have the ability to extract metals from the soil through their deep roots, combined with their commercial prospects, making them an ideal candidate as a profit-yielding crop for phytoremediation purposes. Therefore, a comprehensive review is needed for a better understanding of the morphology and phytoremediation mechanism of four commonly bast fiber plants, such as hemp (*Cannabis sativa*), kenaf (*Hibiscus cannabinus*), jute (*Corchorus olitorius*) and Flax (*Linum usitatissimum*). This review article summarizes the existing research on the phytoremediation potential of these plants grown in different toxic pollutants such as Lead (Pb), Cadmium (Cd) and Zinc (Zn). This work also discusses several aids including natural and chemical amendments to improve phytoremediation. The role of these amendments in the bioavailability of contaminants, their uptake, translocation and bioaccumulation, as well as their effect on plant growth and development, has been highlighted in this paper. This paper helps in identifying, comparing and addressing the recent achievements of bast fiber plants for the phytoremediation of heavy metals in contaminated soil.

## 1. Introduction

Industrialization includes the rapid growth in manufacturing and production as well as technological changes. Growth is required for better productivity, an increase in the standard of living, growth in population, urbanization and more. The rise in urbanization is also expected to go up to 60% by 2030. However, this transformation is causing a drastic change in Earth’s ecosystem, negatively impacting the environment with air pollution, topsoil contamination, groundwater contamination and water pollution. Industrial wastes are more toxic compared to municipal wastes because of the presence of oil, grease, heavy metals, phenols, ammonia and more [1]. Emissions from mining, power plants and refineries are some of the major sources of hazardous toxic chemicals that pollute the environment.

Soil pollution is characterized as the accumulation of persistent toxic compounds, chemicals, salts, radioactive materials, or disease-causing agents, which adversely affect plant growth and animal health in soils. This pollution decreases the quality of the crop as the effect of using of pesticides and chemical fertilizers. Exposure to toxic and dangerous chemicals can increase the health risks to people living nearby and on polluted land. For example, heavy metals can enter humans’ bodies through food, water, air and bioaccumulation over a period of time [2]. This could lead to acute and chronic illness in the central nervous system and peripheral nervous system [3]. Moreover, the toxic effects of heavy metals can cause an imbalance in the ecosystem of the soil. Heavy metals in soils exist in four different forms: dissolved ions, organic complexes, exchangeable ions and precipitates [4]. These compositions are dangerous because they tend to bioaccumulate in plant tissues. Metals such as zinc (Zn), nickel (Ni), manganese (Mn), iron (Fe) and copper (Cu) do contribute their importance in plant growth and help physiological processes such as the electron transfer system in photosynthesis. Other metals such as cadmium (Cd), arsenic (As), chromium (Cr), mercury (Hg) and lead (Pb) do not carry any known biological roles in plants. However, an excessive amount of heavy metal will affect biological and biochemical processes negatively by restraining growth and lowering the chlorophyll content of the plants. For instance, a plant with high lead concentrations fastens the production of reactive oxygen species (ROS), causing lipid membrane damage that ultimately leads to damage of chlorophyll and photosynthetic processes and suppresses the overall growth of the plant [5]. 

Heavy metal contamination in soil has a negative impact on the environment, especially on soil quality and plant growth. Once the plant is saturated with heavy metal, the plant dies due to the interruption in photosynthesis and protein synthesis. Elimination of heavy metals is difficult as it is irreversible and remediation needs to be done. Remediation can be divided into in-situ and ex-situ remediation. In-situ remediation is a process of remediation that does not require transport of contaminated soil to off-site treatment facilities. Ex-situ remediation, on the other hand, is the remediation technique that requires excavation of contaminated soil to an off-site treatment facility [4]. This process requires additional costs. However, the treatments are controlled and accelerated and provide better results in a shorter time. Examples of in-situ remediation are surface capping, encapsulation, electro-kinetics, soil flushing, immobilization, phytoremediation and bioremediation. Examples of ex-situ remediation techniques are landfilling, soil washing, solidification and vitrification [4]. 

Phytoremediation is a cost-effective remediation technique with ecological benefits and high public acceptance. This method is scientifically proven for the remediation of contaminants with the only limitations being the time-consuming process and the possibility of adverse effects on living beings due to biomagnification. This limitation can be overcome using non-edible commercial plants that have rapid growth rates and are easy to maintain. With these characteristics, a bast fiber plant with various plant parts is a good option for phytoremediation. They are also used in the production of a variety of products, such as paper, textiles, wrapping materials, rope, strings, baskets and so on, which will improve the socioeconomic status of people who live in contaminated areas or who use contaminated lands for agricultural purposes. Bast fibre, also known as phloem fibre, is a type of plant fibre derived from the phloem or bast that surrounds the stem of certain dicotyledonous plants. Bast fibres plants can be obtained from either cultivated herbs such as Flax, Hemp and Ramie, or from wild plants such as linden, wisteria and mulberry. The physical properties of different bast fibers that possess a series of characteristics: (1) ability to accumulate metals preferable in the above parts, (2) tolerance to accumulated metal concentrations, (3) production of high biomass and (4) not consumable by humans and animals, making them suitable for use in phytoremediation [6,7].

**Figure 1:**
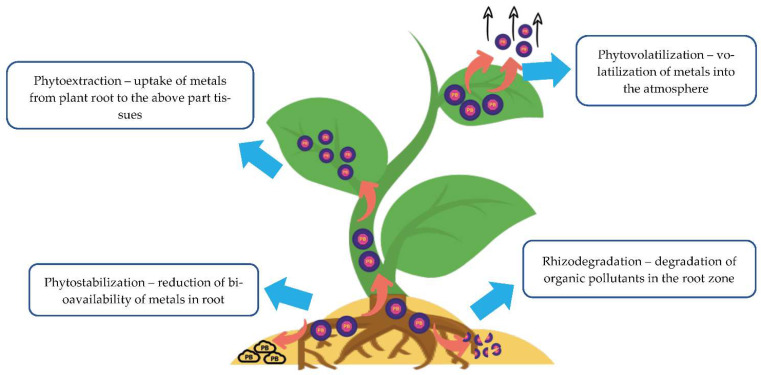
Mechanisms involved in the phytoremediation process.

It is also crucial to understand that edible plants are not appropriate for phytoremediation because they may affect the health of humans or animals once they are consumed [8]. Therefore, fiber crops are said to be the best fit for phytoremediation. This is because fiber plants involve a cycle of planting and harvesting, which help to reduce the heavy metal contamination in the soil over time, and the harvested fiber is used to manufacture biomaterials such as paper and textiles. In this case, it does not enter the food chain and affects the environment negatively, such as harming humans or animal health. Apart from that, different plants have different methods for the removal and accumulation of heavy metals (Figure 1). For example, some plants can stabilize or decrease the mobility of the pollutants in the soil through accumulation in the roots through root hairs to stop contaminants’ run-off, bulk erosion and air-borne transport [9]. Other plants may be involved in the process of plant uptake and release into the atmosphere through transpiration, which is known as phytovolatilization. Many phytoremediation processes are possible through better relationships in between plants, microbes, soil and contaminants. These different processes of phytoremediation perform different management options for a better end product to the environment [6].

This paper discusses the potential of four commonly used bast fiber plants namely *Cannabis sativa* (Hemp), *Hibiscus cannabinus* (Kenaf), *Corchorus olitorius* (Jute) and *Linum usitatissimum* (Flax) for phytoremediation of selective heavy metals, such as cadmium (Cd), lead (Pb) and zinc (Zn) from contaminated soil. The main goal of this paper is to provide references for suitable bast fiber plants for heavy metal treatment. In addition, this review summarises these plants’ ability to accumulate heavy metal elements and reveals their potential for use as phyotoaccumulators or phytostabilizers via their uptake mechanisms. This emerging technology can be improved with natural and chemical amendments that make heavy metals bioavailable and soluble.

## 2. Bast Fiber Plants

### 2.1. Morphology and Characteristics of Bast Fiber Plants (Hemp, Kenaf, Jute and Flax)

Bast fibre is a natural fibre derived from the bast environment of certain dicotyledonous angiosperm plant stems. It is made up of cellulose and hemicellulose combined with a lignin or pectin mixture. In this paper, the potential of four different fiber plants from various places in the uptake of heavy metals from contaminated soil was highlighted. The four fiber plants are Hemp (*Cannabis sativa*), Kenaf (*Hibiscus cannabinus*), Jute (*Corchorus olitorius*) and Flax (*Linum usitatissimum*) (Table 1).

Hemp is a member of the Cannabaceae plant family, and the fibre derived from this plant is one of the strongest forms of natural fibre [10]. It has the potential to be an environmentally friendly and a highly sustainable crop if it is well managed. On the other hand, Kenaf and Jute come from the same family of Malvacea. Kenaf is a non-wood fiber that can be used for reinforcement and it is the world’s third traditional crop after wood and bamboo, which originate in Asia and Africa [11]. Jute fibers are totally biodegradable as it is partially wood [12]. Flax is a member of the Linaceae family of plants, and because its exceptional qualities, Flax fibres are significant raw materials for textiles [12]. Flax and Hemp do not have much difference because they are both cellulose fibers, except that Hemp has ten chromosomes (2n = 20), whereas Flax has 15 pairs of chromosomes (2n = 30) [13]. Kenaf and Jute are woody-stemmed herbaceous dicotyledons grown in the tropics and subtropics. 

### 2.2. Application of Bast Fiber Plants (Hemp, Kenaf, Jute and Flax)

Fiber plants are useful not only for phytoremediation but also in a variety of other fields in the world (Table 2). The bast fibre of hemp plants is used in the automotive industry and textile industry, whereas the whole plant part is used for feedstock and biofuel. Hurds are used for paper production and as a building material such as fiberglass. Hemp oil from the seeds is used in shampoos, soaps and bathing gels. The seeds are also applicable in the food industry as hemp milk and are used as a salad dressing. Technical commercial products such as oil paints, ink and coatings are also produced by these plants [18]. However, the usage of the plants is based on the quality of the hemp. On the other hand, Jute is the second most important fiber plant in the world, and it is also one of the cheapest-grown fiber plants in the tropical region. It is traditionally used to manufacture packaging materials such as sacking, ropes, twines and carpet-backing cloth. Moreover, diversified Jute is also used in the production of home textiles, composites, geotextiles, paper pulp, technical textiles, chemical products, handicrafts and fashion accessories. The woody central core is used as a rural building material for fences, fuel and for charcoal-making. In the Philippines, the leaves of Jute are used to treat headaches [19].

Kenaf also has its own uses and one of them is paper production. Kenaf paper is stronger and more resistant to yellowing compared wood paper and it requires fewer bleaching agents. Furthermore, Kenaf seeds produce edible oil, which is one of the best cooking oils. Dried Kenaf leaves are consumed as a vegetable in some countries because they contain 30% crude protein. The fruit of Kenaf helps in lowering blood pressure and the presence of vitamin C and antioxidants in Kenaf help in fighting some diseases. Kenaf will be used in new applications such as medicines, textiles, natural fiber compounds and environmental cleaning [20]. Flax is used for fruit, medications and textiles and has therefore been used for food processing. It has been of considerable significance for human civilization and growth for more than 8000 years. For many years, Flax was commonly used for the manufacture of fabrics, although nowadays, oil is the main source in production [21].

### 2.3. Case Study on Phytoremediation of Heavy Metals Pb, Zn and Cd by Bast Fiber Plants

In this study, Hemp (*Cannabis sativa*), Kenaf (*Hibiscus cannabinus*), Jute (*Corchorus olitorius*) and Flax (*Linum usitatissimum*) were chosen to compare their potential for phytoremediation of Pb, Cd and Zn in the soil (Table 3). Hemp plants were harvested from agricultural activities with acidic soil value. The concentrations of these metals were higher in the root than in the leaves and shoots. Hemp can tolerate high concentrations of Zn and most of the Zn absorbed is retained in the roots [26]. The uptakes of these heavy metals are significantly influenced by the pH of the soil. This statement is supported by the study caried out by Gray et al. [27], where the results showed that increasing the pH will cause a significant reduction in the concentration of cadmium in clover, lettuce, carrot and ryegrass. 

Research conducted by Nizam et al. [28], highlighted that the concentration and uptake of Pb by the shoot were significantly higher than the root in the Kenaf plant. Most of the varieties grown in Pb contaminated soil accumulated more Pb in shoots than roots, indicating that Pb was easily transported from root to shoot in Pb-contaminated soil. This could be related to the Pb content and its relationship with other essential ions during nutrient uptake. Other studies by Shehata et al. [8] mention that Kenaf plants were irrigated with wastewater, and sulfur soil addiction with humic acid was used as foliar spraying and it showed the significant highest accumulation of cadmium, which was 0.87 mg/kg in the roots and 0.36 mg/kg in the shoots. They noticed that humic acids are the most active components in soil and compost as it improves the uptake and accumulation of heavy metals in the tissues’ plant [29]. Cecília et al. [30], studied the phytoremediation of zinc and the results showed that Kenaf is able to absorb 233 mg/kg of zinc in the roots and 264 mg/kg in the shoots. 

Furthermore, the studies about phytoremediation in untreated industrial wastewater from textile factories by Ahmed and Slima [31] show that there was very high concentration of Cd in the roots with 261.83 mg/kg and 41.35 mg/kg in the shoots of the Jute. In contrast, the concentration of Pb in the roots was 367.83 mg/kg, whereas in the shoots it was 370.43 mg/kg. This finding shows that the nutrients in the roots and shoots were decreased significantly because of contamination stress. Lead (Pb) is a toxic heavy metal that can inhibit plant growth, seedling development and root elongation [32]. They also state that Flax is a fibre plant that is suitable for growing in industrially polluted areas because its root system removes significant amounts of heavy metals from the soil and can be used as a potential crop for cleaning the soil of heavy metals [33]. Hosman et al. [34], studied the bioremediation potential of Flax under different concentration of Pb, Cd and Zn. The average ability of the Flax plant to remove heavy metals from soil was 49% for Cd, 68.6% for Pb and 71.76% for Zn. Following that, the highest accumulation of Cd was found in the root, whereas the highest accumulation of Pb and Zn was found in the capsule. He also reported that by increasing the metal concentration in the soil, there was a gradual increase in metal uptake in the Flax plant. Several phytotoxicity effects were observed when these metals exceeded the endogenous level [35].

### 2.4. Enhancing Phytoremediation of Heavy Metals of Bast Fiber Plants by Chemical and Microbiological Amendments

The phytoremediation potential of bast fiber plants can be increased by using chemical amendments in the soil and microbial enhancement through inoculation in the roots of plants. Chemical amendments play a key role in compensating for the relatively low heavy metal availability in soil, and it helps the plants’ uptake and translocates metals toward the shoot [43]. Previous studies have reported that various chelators are employed to increase the solubility of metals in soil, including 1,2-cyclohexane-diaminetetraacetic acid (CDTA), ethylene glycol tetraacetic acid (EGTA) and diethylene-triaminepentaacetic acid (DTPA) [44,45,46]. One of the most effective chelating agents is ethylenediaminetetraacetic acid (EDTA), which can increase the solubility, absorption and complexation of metals (including Pb ions in soil) [5,47,48,49]. Furthermore, metal-EDTA complexes may form and function to significantly boost Pb ion absorption by plant roots and translocate them to shoots [50]. Hasan et al. [51] reported that metallothioneins produced by certain genes could withstand conditions where metal stress is present in the environment. Furthermore, this metal-binding protein with low molecular weight can facilitate the metal ion into the plant cells and translocate them via the xylem. In phytoremediation technologies, the addition of nutrients to plants may results in healthy plant growth with the development of flowers, leaves and branching of the root system, and can thus increase the level of uptake contaminant in the study area. However, an excessive amount of nutrients given to the plants can result in a significant reduction in plant growth. This symptom is known as nutrient toxicity. In a nutrient-enriched environment, the bioavailable fraction of metals may be reduced because of the binding to the nutrient anions. The uptake of heavy metals in plants may also be affected by competition since nutrient cations compete with the metal for uptake sites [52]. Thus, the uptake of the metal under investigation decreases with an increasing concentration of nutrients. However, a generous availability of nutrients promotes plant growth, which in turn creates an increasing number of uptake sites for metal in plants. This may increase the uptake as well as the metal concentrations in plants. 

Interactions between plants and microbes are crucial factors in determining the efficiency of phytoremediation [53]. These interactions are implicated to play an essential role in plant metal uptake. The beneficial microbes associated with plants directly improve the efficiency of the phytoremediation process by altering metal accumulation in plant tissues and indirectly by promoting shoot and root biomass production. Whiting et al. [54], reported that the biomass and zinc concentration in the shoots of *Thlaspi caerulescens* has been increased with the presence of rhizospheric bacteria. These bacteria can promote plant growth by inhabiting the plant roots [55] and are known as plant growth-promoting rhizobacteria (PGPR) [56]. The generation of phytohormones, specialized enzymatic activity, nitrogen fixation in the atmosphere and pathogen-depressing chemicals such sidephores and chelating compounds all contribute to the role of PGPR in promoting plant growth [57]. Sidephores and chelating compounds have been shown to promote plant growth even in the presence of heavy metals [58]. 1- aminocyclopropane- carboxylic acid deaminase is another plant growth-promoting compound that has been studied in relation to heavy metals (ACC deaminase). ACC is an intermediate of ethylene produced by stressed plants, and bacteria that produce ACC deaminase can reduce ethylene levels in plants, promoting plant growth [59]. 

**Figure 2:**
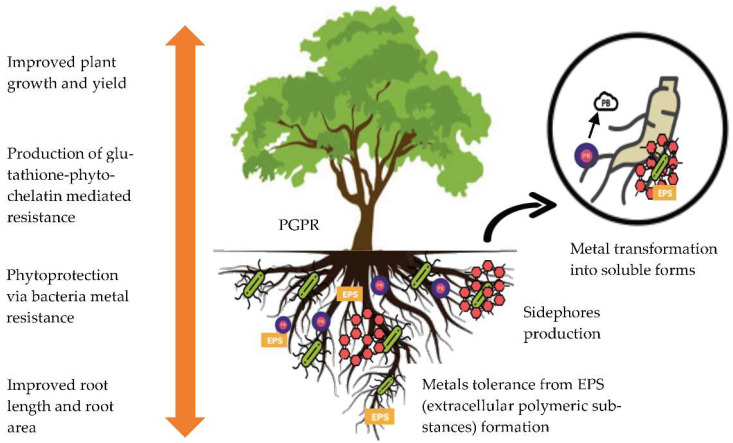
The mechanism of plant-microbe association that supports metals phytoremediation.

In another study, Belimov et al. [60] discovered that bacteria containing ACC deaminase can improve plant growth in metals-polluted conditions. Meanwhile, Braud et al. [61], studied the phytoextraction of agricultural Cr and Pb with sidephore- producing bacteria, and highlighted that the inoculated Maize plant with bacteria enhanced the bioavailability and uptake of Cr and Pb. Khan et al. [62], investigated the (Ni) accumulation of mycorrhizal and non-mycorrhizal Flax plants at various concentrations of Ni, i.e., 0, 250, 350 and 500 ppm. He reported that the accumulation of metals was higher in mycorrhizal than in non-mycorrhizal plants. Additionally, mycorrhizal plants showed noticeably greater growth and development than non-mycorrhizal plants. The production of phytohormones by Arbuscular Mycorrhizal Fungi (AMF) can improve nutrient and water uptake as well as improve metal bioavailability and aid in the phytoremediation process [63]. Figure 2 shows the mechanism of plant-microbe association that supports metal phytoremediation.

### 2.5. Molecular Mechanisms Involved in Microbial Resistance to Heavy Metals

Microorganisms have been involved in the mechanisms of adapting to heavy metals either in water or soil [64]. Some metals, such as copper, nickel and cobalt, are given to microorganisms as micronutrients for use in redox processes, to stabilise molecules through electrostatic interactions, to act as components of various enzymes and to regulate osmatic pressure. Otherwise, non-essential metals are recognized as having little nutritional value and may be toxic to microorganisms. To overcome the toxicity value, there are six metal mechanisms that exist in the microorganism, including the exclusion of the permeability barrier, intra- and extra-cellular sequestration, active transport efflux pumps, enzymatic detoxification and reduction in the sensitivity of cellular targets to metal ions.

#### 2.5.1. Metal Exclusion by Permeability Barrier

The metal exclusion by the permeability barrier involves changes in the cell wall, membrane or envelope of microorganisms. This mechanism is an attempt by the organism to protect metal-sensitive and essential cellular components. Previous research has shown that bacteria form an extracellular polysaccharide coating that has the ability to bio-absorb heavy metal ions and prevent them from interacting with vital cellular components [65]. These bacteria’s exopolysaccharide coating may provide sites for metal cation attachment [65]. For example, there are several strains of bacteria that demonstrated the ability to bind metals extracellularly, such as *Klebsiella aerogenes*, *Pseudomonas putida* and *Arthrobacter viscosus*. According to Scott and Palmer, [65] a protective layer of exopolysaccharide improves the survival of *K. aerogenes* strains in Cd (II) solutions. When compared to strains without their protective layer, these strains show a two-fold increase in Cd (II) accumulation. This protective layer appears to help reduce toxicity by preventing metal ion uptake and keeping metal ions away from sensitive cellular components.

#### 2.5.2. Active Transport of the Metals Away from the Microorganisms

One of the largest categories of metal resistance systems is an active transport or efflux system by microorganisms. These methods involve the cytoplasmic export of harmful metals. These processes may be plasmid- or chromosomal-encoded. Normally, nutrient transport systems allow non-essential metals to enter the cell, but they are quickly expelled. These efflux mechanisms are extremely selective for the cation or anion they export and can be either non-ATPase or ATPase-linked [66]. *Bacillus subtilis, S. aureus* and *E. coli* [67] are only a few of the microorganisms that have shown resistance to Cd (II). The plasmid-encoded cad system in *S. aureus*, as reported by Smith and Novick, [68] is the best-characterized Cd (II) resistance efflux. Early research shows that there are two distinct plasmid-mediated Cd (II) resistance mechanisms. The first has single cad loci (cadA) responsible for conferring resistance, and the second has two loci cadA and cadB [68]. cadA shares strong amino acid sequence homology with P-class ATPase, which functions as an ion pump [69]. CadA proteins have six major domains that work together to form a pump that removes Cd (II) from the cell’s interior. An outer cytoplasmic metal binding region, a transmembrane domain and a transduction ‘funnel’ that may move bound Cd (II) to the membrane surface comprise the domain.

#### 2.5.3. Intracellular and Extracellular Sequestration of Metals by Protein Binding

The accumulation of metals within the cytoplasm to avoid exposure to essential cellular components is known as intracellular sequestration. Metals that are commonly sequestered include Cd (II), Cu (II) and Zn (II). Otherwise, extracellular sequestration is the mechanism involved in the secretion of large amounts of glutathione. The production of metallothionein by *Synechococcus* sp. is an intracellular sequestration [70]. Two genes, smtA and smtB, make up *Synechococcus* sp. metal’s resistance system. A metallothionein that binds Cd (II) and Zn is encoded by smtA. (II). High levels of Cd (II), Zn (II) and Cu (II) stimulate these genes, which are then suppressed by the smtB gene product. The smtB protein functions as a transacting transcriptional repressor, inhibiting the expression of smtA and the synthesis of metallothionein [70]. For extracellular sequestration in yeast, Murata et al. [71], reported that Saccharomyces *cerevisiae* may reduce the absorption of Ni (II) by excreting a gluthathione. Gluthathione binds with great affinity to heavy metals and carrying the methyglyoxal resistance gene and demonstrates the ability to form extracellular metal-gluthathione complexes in metal rich media [71].

#### 2.5.4. Enzymatic Detoxification of Metals to a Less Toxic Form

Mercury resistance is a prime example of an enzymatic detoxifying system in bacteria. Mercury is classified as a toxic metal because it binds to and inactivates essential thiols found in enzymes and proteins. Microorganisms such as Gram-positive (*S. aureus, Bacillus* sp.) and Gram-negative bacteria (*E. coli, P. aeuruginosa and Thiobacillusf errooxidans*) have been shown to demonstrate resistance to the Hg (II) (*mer*) resistance operon. This operon not only transports and self-regulates resistance, but it also detoxifies Hg (II) [72]. The same side of these genes also encodes the creation of a periplasmic binding protein and membrane-associated transport proteins. Hg (II) in the immediate surroundings is gathered by the periplasmic binding protein and transported to the cytoplasm by transport proteins for detoxification.

#### 2.5.5. Reduction in Metals Sensitivity of Cellular Targets

Rouch et al. [73], demonstrated that some microorganisms can adapt to the presence of hazardous metals by changing how sensitive some vital cellular components are, offering some degree of natural defense. Protection is achieved either by boosting the production of a specific cellular component to prevent a metal inactivation or by mutations that reduce sensitivity without changing basic function. The microorganism may potentially defend itself by creating metal-resistant parts or an alternative pathway to get around vulnerable parts. This adaptation was discovered in *E. coli* after exposure to Cd (II) [73]. Rouch et al. [73], highlighted that the longer an organism is exposed to Cd, the shorter its growth at the lag phase is (II). The extended lag phase is thought to be caused by a period of induction of DNA repair mechanisms. Natural resistance can develop as a result of normal cellular functions that provide the organism with a basic level of tolerance to heavy metals [73].

## 3. Advantages and Limitations of Phytoremediation

As mentioned earlier, phytoremediation is a promising method for cleaning up heavy metal-contaminated soils. Despite the numerous challenges, phytoremediation is regarded as a green remediation technology with enormous potential. The main advantages of this method are cost effectiveness, eco-friendliness and practicality compared to other mediation technologies. However, there are some limitations that need to be addressed in this process. This includes huge funds expenditure and human resources as well as favorable weather and climatic conditions for plants. The advantages and limitations of phytoremediation are described in detail in Table 4.

## 4. Summary

Global trends toward sustainable development have brought phytoremediation as one of the emerging technologies for the decontamination of heavy metals in soil. Bast fiber plants are very promising candidates since they show tolerance to toxic trace elements in soils, have fast-growing and yield high biomass, have low maintenance, and are well known in the industrial sector. Based on the heavy metal content results in the fiber crops studied, the following conclusions can be drawn:

1. Heavy metal accumulation in bast fiber plants is clearly showed in vegetative and reproductive organs. Hemp (*Cannabis sativa)* is the crop that most strongly accumulates Zn followed by Kenaf (*Hibiscus cannabinus)*, Jute (*Corchorus olitorius),* and Flax (*Linum usitatissimum)*. It is notable that Jute is more tolerant and best uptake potential for Cd as compared to others crops.
2. It is reported that the distribution of heavy metals Pb, Zn, and Cd is selective to roots as compared to shoot for all bast fiber plants studied.
3. It is suggested that Hemp, Kenaf, and Jute are suitable species for soil remediating of heavy metals Pb and Zn. Therefore, these species can be successfully cultivated for phytoremediation purposes since their root system can remove significant amounts of heavy metals from the soil.

## Figures and Tables

**Table 1:** Morphology and specifics characteristics of bast fiber plants (Hemp, Kenaf, Jute and Flax).

Fiber Plants		Morphology				
	Roots	Stems	Leaves	Flowers	Seeds	Reference
Hemp (*Cannabis sativa*)	Root system is well developed with depth of about 1 to 1.5 m	The stems are normally hollow with diameter ranging from 5 to 25 mm. The base and top stem have different diameters. Mature plant reaches up to 5 m	The first true leaves are single leaflets; later leaves become palmate compounds. The second leaf pair consists of three leaflets per leaf, the third leaf pair has five leaflets per leaf, and so on, up to eleven leaflets per leaf	Male flowers and female flowers available. Female flowers are more compact	Hemp seeds are achenes seeds. Seeds are ellipsoid in shape, 2 to 7 mm long and 2 to 4 mm wide in diameter. Seeds vary in colour from light brown to dark green	[14]
Kenaf (*Hibiscus cannabinus*)	It has a prolific root system with a long taproot and extensive lateral roots	It mainly has unbranched stems and grows up to 4.5 m tall	Young leaves are simple and entire. Divided leaf can produce 3 to 10 entire young leaves prior to the first divided leaf	It produces large showy, light yellow, creamy coloured flowers that are bell-shaped and widely open. The flowers are solitary, short-stalked and auxiliary and are 8 to 13 cm in diameter with 5 petals, 5 sepals and numerous stamens	The seeds are normally brown with 6 mm long and 4 mm wide. The seeds of Kenaf are produced by the fruits, known as fruit capsules in 1.9 to 2.5 cm long and 1.3 and 1.9 cm in diameter with many seeds, around 20 to 26	[15]
Jute (*Corchorus olitorius*)	It has an extensive lateral branching and deep tap root system	The height range of the Jute plant is between 2 and 4 m. The stems are about 1 to 2 cm in diameter with few branches. The colour of the stem, petiole and leaf varies.	The leaves are edible with a bitter taste. Leaves are usually 6–10 cm long and 3.5–5 cm broad	It consists of small pale-yellow flower, bracts lanceolate, 2 to 3 cm wide, sepals 3 mm long and petals are 5 mm long	Seeds are greyish- black and angled	[16]
Flax (*Linum usitatissimum*)	It has short and branched tap root that can extend to a depth, of1 m, with side branches spreading to 30 cm	It has one main stem, but two or more branches (tillers) may develop from the base when plant density is low or with high soil nitrogen levels	The leaves are normally small and lance- shaped	The flowers parts are normally in units of five and can range from a dark to a very light blue, white or pale pink	The seeds are flat, oval and pointed at one end. Normally the seeds are covered in mucilage, giving it a high shine	[17]

**Table 2:** World countries ranking of producing fibre plants.

Types of Fiber Plants	Hemp (*Cannabis sativa*)	Kenaf (*Hibiscus cannabinus*)	Jute (*Corchorus olitorius*)	Flax (*Linum usitatissimum*)
Ranking				
1	China	India	India	Russia
2	Canada	China	Bangladesh	Canada
3	United States of America	Thailand	China	Kazakhstan
4	France	Brazil	Uzbekistan	China
5	Chile	Vietnam	Nepal	United States
6	North Korea	Cuba	South Sudan	India
7		Indonesia	Zimbabwe	
8		Pakistan	Egypt	
9		Pakistan	Vietnam	
10		Cambodia	Bhutan	
References	[22]	[23]	[24]	[25]

**Table 3:** Heavy metal concentration in Bast Fiber Plants. Listed tissues represent those with the highest concentration of metals in the roots, leaves and shoots.

Types of Fiber Plants	Metals	Concentration (mg/kg^−1^)			Reference
		Roots	Leaves	Shoots	
Hemp (*Cannabis sativa*)	Pb	38.2	16.5	23.5	[33]
	Pb	14.6	2.22	2.07	[36]
	Cd	2.82	0.23	0.37	[36]
	Cd	1.03	0.55	0.98	[33]
	Zn	688.6	323.1	156	[36]
	Zn	66.8	40.0	54.5	[33]
Kenaf (*Hibiscus cannabinus*)	Pb	2.43	-	8.9	[28]
	Pb	329.66	-	867.55	[37]
	Cd	0.87	-	0.36	[8]
	Cd	0.25	-	0.14	[38]
	Zn	233.0	-	264.0	[30]
	Zn	114	65	-	[39]
	Zn	377.78	133.33	-	[40]
Jute (*Corchorus olitorius*)	Pb	21.74	-	-	[41]
	Pb	367.83	370.43	-	[31]
	Cd	163	-	48	[31]
	Cd	261.83	41.35	-	[42]
	Zn	148.53	151.42	-	[42]
Flax (*Linum usitatissimum*)	Pb	104.4	14.5	30.2	[33]
	Pb	310.56	-	-	[34]
	Cd	13.06	-	-	[34]
	Cd	8.69	1.62	7.27	[33]
	Zn	255.71	-	-	[34]
	Zn	211.8	32.6	62.9	[33]

**Table 4:** The advantages and limitations of the phytoremediation process.

Advantages	Limitations	Reference
It is cost-efficient	It takes longer time to achieve the results as it is a slow process	[74,75]
Soil properties will not be affected during the process of phytoremediation, as it is environmentally friendly	The toxins, pH and concentration of contaminants must be below the plant’s tolerance level	
Applicable for large, contaminated areas	Cannot be carried out in a medium with excessive concentration of contaminants suitable for shallow contamination (within the rooting zone) at non-excessive concentrations	[76]
Helps to reduce the possibility of soil erosion and prevent the metals in the affected area from leaching	Possibility of high toxins entering food chain because of poor management	
Can be used for both in situ and ex situ applications	Only suitable for shallow contamination, which means until the depth of the root	[77]
Has the potential to be a permanent treatment in treating a wide range of contaminants	The remediated plant biomass could be dangerous as it contains hazardous wastes	

## Data Availability

Not applicable.
